# Augmented feedback modes during functional grasp training with an intelligent glove and virtual reality for persons with traumatic brain injury

**DOI:** 10.3389/frobt.2023.1230086

**Published:** 2023-11-22

**Authors:** Mingxiao Liu, Samuel Wilder, Sean Sanford, Michael Glassen, Sophie Dewil, Soha Saleh, Raviraj Nataraj

**Affiliations:** ^1^ Department of Biomedical Engineering, Stevens Institute of Technology, Hoboken, NJ, United States; ^2^ Movement Control Rehabilitation (MOCORE) Laboratory, Altorfer Complex, Stevens Institute of Technology, Hoboken, NJ, United States; ^3^ Center for Mobility and Rehabilitation Engineering Research, Advanced Rehabilitation Neuroimaging Laboratory, Kessler Foundation, NJ, United States

**Keywords:** traumatic brain injury, virtual reality, motor rehabilitation, sensory feedback, hand grasp, physical therapy

## Abstract

**Introduction:** Physical therapy is crucial to rehabilitating hand function needed for activities of daily living after neurological traumas such as traumatic brain injury (TBI). Virtual reality (VR) can motivate participation in motor rehabilitation therapies. This study examines how multimodal feedback in VR to train grasp-and-place function will impact the neurological and motor responses in TBI participants (*n* = 7) compared to neurotypicals (*n* = 13).

**Methods:** We newly incorporated VR with our existing intelligent glove system to seamlessly enhance the augmented visual and audio feedback to inform participants about grasp security. We then assessed how multimodal feedback (audio plus visual cues) impacted electroencephalography (EEG) power, grasp-and-place task performance (motion pathlength, completion time), and electromyography (EMG) measures.

**Results:** After training with multimodal feedback, electroencephalography (EEG) alpha power significantly increased for TBI and neurotypical groups. However, only the TBI group demonstrated significantly improved performance or significant shifts in EMG activity.

**Discussion:** These results suggest that the effectiveness of motor training with augmented sensory feedback will depend on the nature of the feedback and the presence of neurological dysfunction. Specifically, adding sensory cues may better consolidate early motor learning when neurological dysfunction is present. Computerized interfaces such as virtual reality offer a powerful platform to personalize rehabilitative training and improve functional outcomes based on neuropathology.

## 1 Introduction

Trauma to the brain can severely impair motor function to perform activities of daily living (Colantonio et al., 2004). For affected individuals, rehabilitation of hand function, especially reaching and grasping, is critical for environmental access (Chaabani et al., 2014). Physical therapy is a primary option to rehabilitate hand function; however, traditional therapy involves intensive and repetitive movement training (Connell et al., 2018). Feelings of rigor during training are naturally detrimental to efficient gains in function; thus, methods fostering greater engagement are needed to overcome the monotony of physical practice (Lohse et al., 2013). Newer approaches to physical therapy are seeking to utilize advanced technologies, such as virtual reality (VR) (Howard, 2017) and instrumented wearables (Simone et al., 2007), to motivate participation in therapy.

Computerized technology, especially virtual reality, is increasingly employed in motor rehabilitation to facilitate greater motivation and to provide customizable training options, including enhanced feedback (Merians et al., 2006). Computerized interfaces can provide robust movement guidance (Gorgey, 2018) and leverage cognitive elements of physical training that can accelerate motor learning after neurological traumas (Mulder and Hochstenbach, 2005). Given their vast programmable features, virtual reality environments are well suited to personalize rehabilitative training that maximizes user engagement and functional outcomes based on neural processes (Holden and Todorov, 2002). Integrating advanced technologies with motor rehabilitation creates a user-computer interface that can motivate with colorful and immersive environments while also providing real-time guidance using enhanced sensory-driven feedback to facilitate motor recovery (Mulder and Hochstenbach, 2005). Thus, virtual environments can optimize motor learning by manipulating training conditions, e.g., guidance cues, for a given user profile, e.g., pathological features, to broadly affect motivational, cognitive, motor, and sensory learning mechanisms (Levin et al., 2015).

Augmented feedback with sensory cues informing individuals about performance achievements or errors during training is proven to enable motor learning (Sigrist et al., 2013). Augmented feedback activates sensory modalities (e.g., visual, audio, haptic) to guide performance during training (Sigrist et al., 2013). With “multimodal” augmented feedback, more than one sensory modality is activated concurrently to hasten motor learning trajectories by broadening the areas of neural activation and exceeding neural activation thresholds earlier during repeated practice (Sigrist et al., 2013; Seitz and Dinse, 2007). Thus, multimodal feedback in VR motor rehabilitation training is a promising approach to recovering motor function after neurological traumas. Our lab has shown how motor performance is sensitive to features in augmented feedback (Sanford et al., 2020) using computerized interfaces for either motion (Sanford et al., 2021) or myoelectric control tasks (Sanford et al., 2022; Walsh et al., 2021).

Still, it remains unclear if persons with neurological damage, such as traumatic brain injury (TBI), respond similarly to augmented feedback approaches as neurotypicals. Given disturbed brain connectivity after TBI (Hayes et al., 2016), the ability to process sensory cues (Folmer et al., 2011) and subsequently apply them with functional capabilities (Ciccarelli et al., 2020) can be compromised. Another potential challenge in utilizing augmented sensory feedback with TBI is a possible deficiency in synchronizing cues with the functional task being practiced (Ghajar and Ivry, 2008). Accurately inferring times of cues relative to task actions is especially critical to ensure motor training with augmented feedback will be effective.

Our lab has previously developed and verified the potential of training with an intelligent glove system capable of providing augmented sensory cues for a functional grasp task while also inducing a sense of agency (Liu et al., 2021). Sense of agency, or perception of control, is a cognitive measure highly associated with motor function (Moore, 2016). Intentional binding is an implicit measure of agency (Moore and Obhi, 2012), which manifests from the compression of one’s perception of the time between a voluntary action and an expected outcome. Our lab has shown positive relationships between implicit measures of agency and movement performance (Nataraj et al., 2020a; Nataraj et al., 2020b; Nataraj and Sanford, 2021; Nataraj et al., 2022) and seeks to leverage such connections for better rehabilitation approaches.

In our training paradigm with the glove system, we facilitate a sense of agency through intentional binding by progressively reducing the delay between the user’s action of a “secure” grasp and the outcome of sensory cues from the onboard modules. The glove system includes onboard force and flex sensors and a processor for an artificial neural network to identify secure grasp, as detailed in (Liu et al., 2021). Participants are cued about their action of securely grasping an object based on sensory-activation modules (visual: LED light, audio: beeper) onboard the glove and then proceed to complete the grasp-and-place task. During training, there is a progressive reduction of the delay between the action and consequential sensory cue to stimulate a perception of greater binding and, therefore, stronger feelings of agency. In our previous study with the intelligent glove system (Liu et al., 2021), we reported that neurotypicals demonstrated improved performance of a grasp-and-place task using “binding” feedback during training compared to no feedback or immediate (no delay) feedback. However, it was unclear if participants with neurological impairment may respond similarly, given potential challenges with discerning timing or processing augmented sensory feedback in VR.

The current study seeks to establish how participants with neurological dysfunction (i.e., TBI) will respond using this glove system when augmented sensory feedback is provided in the following ways: 1) progressively binding feedback to actions during training as done in (Liu et al., 2021), 2) further enhancing the sensory cues through VR, and 3) comparing the effects between providing unimodal (audio only) and multimodal (audio plus visual) cues. Responses in the presence of TBI will be characterized along domains of neural activation (electroencephalography, EEG), functional motor performance, and muscular engagement (electromyography, EMG) and compared against neurotypical responses. We hypothesized that multimodal feedback in VR will support greater neural (EEG) and muscular (EMG) activation and improve performance (reduced motion pathlengths, reduced completion times) of a grasp-and-place task for persons with TBI.

## 2 Materials and methods

### 2.1 Participants

Persons with TBI (*n* = 7) were recruited for a funded study (New Jersey Health Foundation, Research Grant PC 53-19) and tested at Kessler Foundation. These participants signed an informed consent form approved by the Institutional Review Board (IRB) at Kessler. The committee, composed of persons not associated with a given study, reviews and approves all human research studies at Kessler annually. They assure the safety of study participants, patients and healthy volunteers, including the use of clear language in the consent form. These participants were diagnosed as having moderate-to-severe TBI with upper extremity deficits.

Participants with TBI were classified based on the TBI Model Systems National Database (Dijkers et al., 2010), where one of the following criteria must be met: (a) loss of consciousness for 30 min or more; (b) posttraumatic anterograde amnesia for 24 h or more; (c) lowest Glasgow Coma Score (GCS) (Sternbach, 2000) in the first 24 h ≤ 15 (unless due to intubation, sedation, or intoxication); or (d) evidence of significant neurological injury on CT/MRI (e.g., subdural hematoma, cerebral contusion, subarachnoid hemorrhage). Severity was further defined using the following GCS score criteria: mild (14–15), moderate (9–13), or severe (3–8). Injury severity was confirmed from medical records when possible; in the absence of medical records, severity was determined by family member attestations of the length of loss of consciousness/coma.

Another group of neurotypical participants (*n* = 13) was recruited among a pool of students at Stevens Institute of Technology and compensated using funds from the Charles V. Shaefer, Jr. School of Engineering and Science at Stevens. These participants were tested at Stevens after signing an informed consent form approved by the Stevens IRB. The Stevens IRB is composed of members internal and external to the institution, and it reviews and approves all human research studies at Stevens annually. Neurotypical participants did not report nor indicate complications involving cognition or upper extremity function.

Study enrollment did not require participants to undergo clinical function assessments; thus, limited data were available to infer the degree of motor impairment for TBI participants. However, two participants were sampled from a participant pool having undergone timed tasks for the Wolf Motor Function tests (Lin et al., 2009). The average time score was 31 ± 13 s, which correlates to an upper-extremity Fugl-Meyer score of approximately 40 according to (Hodics et al., 2012), which denotes mild-to-moderate motor impairment (Woytowicz et al., 2017). Furthermore, the average maximum voluntary contraction (MVC) for EMG-recorded muscles of the TBI group was 72% ± 40% for the respective muscles of the neurotypical group. MVC exercises included index-thumb gripping (close- and open-grip directions) and wrist flexion-extension. Overall, we presume that TBI participants for this study have relatively high motor function.

### 2.2 Instrumented glove system to detect secure grasp

**FIGURE 1:**
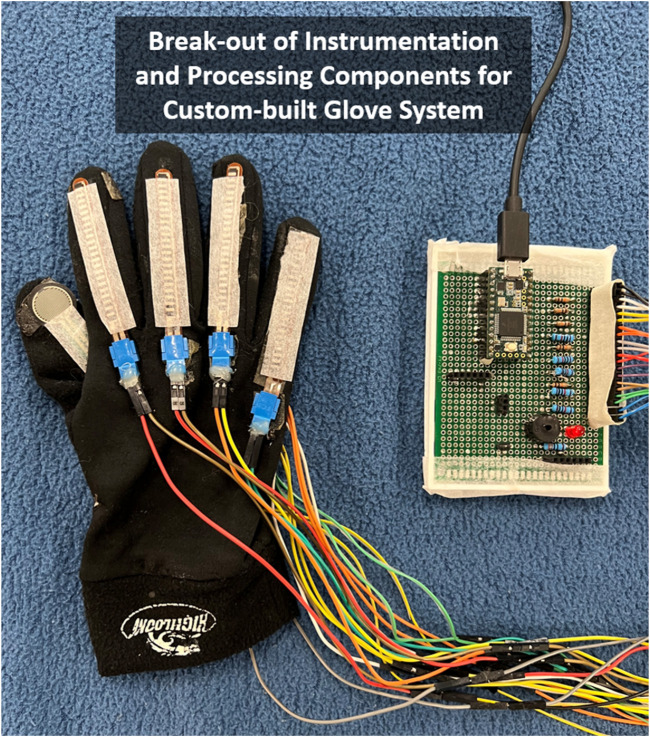
Instrumented glove includes force and flex sensors providing inputs to artificial neural network that predicts when secure grasp on object is achieved and triggers augmented sensory feedback cue. Note: Glove is right-handed shown from dorsal side with thumb inverted to show force-sensitive resistor on palmar side (i.e., thumb-pad).

The glove system hardware (Figure 1) included a compression glove embedded with force (*Interlink Electronics*) and flex (*Spectra Symbol*) sensors across each digit, aligned on the palmar dorsal side, respectively. The sensors were connected to an instrumentation board (*Teensy*) programmed with *Arduino*. The board and wired connections were housed in a custom 3D-printed enclosure with a wrist-strapped mount. Sensory modules onboard the glove included an LED and sound beeper used for visual and audio cues in our previous study (Liu et al., 2021). The function of these sensor modules is now replaced (and enhanced) in this study using VR (details described in Section 2.3). The glove with onboard instrumentation has a mass of under 100 g. API code in *MATLAB* ^®^ (*Mathworks*) read sensor data via serial communication at 40 Hz and was processed on an *Intel* desktop computer (*Xeon* ^®^ 3.20 GHz, 32 GB RAM, *Windows 10 Pro*). The board is programmed to run a trained two-layer feedforward artificial neural network (Neural Network Toolbox, *MATLAB* ^®^, *Mathworks*) to compute (predict) whether hand grasp upon an object is secure (or not) based on inputs from the onboard force and flex sensors. The network creation and training procedures are detailed in (Liu et al., 2021). During each training trial, the glove *1*) identifies the achievement of a “secure” grasp onto an object, *2*) informs the user by activating a feedback module, and *3*) facilitates agency via greater “binding” by progressively reducing the delay (from 1 to 0 s across all training trials) between grasp action and feedback cue. A surgical glove was placed over the sensor glove to ensure a better fit to the hand.

### 2.3 Experimental protocol

All participants donned our custom-built instrumented glove on their self-selected dominant side (left- and right-hand versions available) to perform a functional (grasp-and-place) task for all trials. At Stevens, neurotypical participants wore a 32-channel scalp-surface cap for EEG recording (*USBamp*, *g. tec*) and skin-surface EMG electrodes (*Delsys Trigno*) at hand and forearm muscles. EMG recordings were taken at the following seven muscle sites: flexor carpi radialis (proximal flexor), extensor carpi radialis brevis (proximal extensor), flexor digitorum superficialis (distal flexor), extensor digitorum communis (distal extensor), abductor pollicis brevis (thumb abductor, palmar-side recording), adductor pollicis (thumb adductor, dorsal-side recording). At Kessler, TBI participants wore a 64-channel scalp-surface cap for EEG recording (*actiCHamp Plus*, *BrainVision*) and seven EMG electrodes (*Power Lab/30 Series*) at the same locations as neurotypical participants. Protocols at Kessler and Stevens were identical except for the number of trials collected in each block (explained below).

**FIGURE 2:**
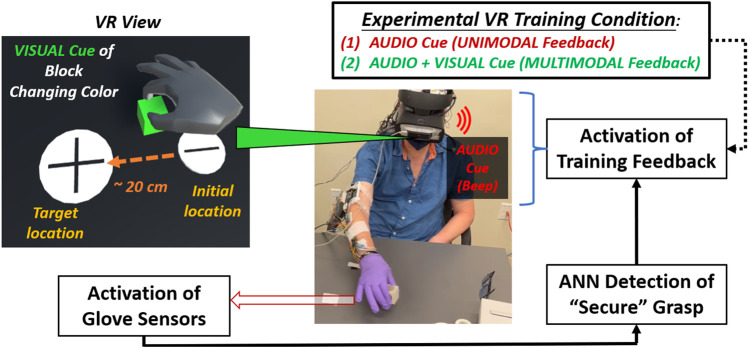
Flow diagram shown for experimental procedure for mixed-mode reality grasp-and-place task. Participant wears instrumented glove (under surgical glove) in grasping and moving cubic object while receiving augmented sensory feedback during training.

The motor task for each trial entailed reaching and grasping a small cubic object, lifting it from an “Initial” location, and then moving and placing the object onto a “Target” location (Figure 2). Participants were asked to grasp the object with a precision pinch, i.e., using index finger and thumb (Nataraj et al., 2014). Participants with TBI were encouraged to adapt their grasp strategy as needed to perform the task successfully. However, all TBI participants could achieve a precision pinch grasp without discernible adaptation. Participants were informed that they were assessed for performance *primarily* on minimizing the object’s motion pathlength and *secondarily* on placing it accurately on the designated target and completing the task promptly.

In adding VR feedback to our glove system, participants experienced mixed-mode reality. They manipulated a real object while viewing a VR environment (*Unity*) through a headset (*HTC Vive*) displaying virtual representations of the object and the gloved hand. These representations were identified and translated into VR using a motion controller (*LEAP*). Calibration procedures were performed to synchronize the positions of the virtual and real cubic objects and have them coincide with the participant’s perspective at the start of each trial. Secure grasp was still detected based on glove sensor inputs to the onboard neural network processor.

In VR, the audio feedback was naturally enhanced when provided through the headset’s earpiece. In addition, the visual cues were enhanced by having the entire virtual object change color (red to green) during secure grasp. Augmented feedback cues about secure grasp were only provided during training trials. During training, participants received augmented sensory feedback upon and during secure grasp in VR. Augmented feedback was delayed upon detecting a secure grasp. However, the delay progressively reduced from 1 to 0 s overall training trials to induce agency through binding (Moore and Obhi, 2012). The earpiece provided *unimodal feedback* as a singular beep (“audio cue”). The beep was short (100 m sec duration) with moderate tone and pitch. For *multimodal feedback*, the virtual object *additionally* changed color (“visual cue”) from red to green. The color change activated concurrently with the audio cue, but it was persistently active during secure grasp and would inactivate (i.e., the virtual object turned red again) upon release of the object. Providing multimodal feedback in this way, i.e., persistent visual cue and audio with a single beep, was most effective (least distracting) to participants based on a series of pilot experiments to validate this training approach with neurotypicals initially (Liu et al., 2021).

In each session, a participant executed three blocks of trials: 1) an initial block of trials without feedback to establish baseline performance (i.e., “pre” training), 2) a block of trials to train with augmented feedback at progressively shorter time delay intervals (1–0 s) after “secure” grasp to induce binding, 3) a block of trials without feedback to determine effects after (i.e., “post”) training. For pre/train/post blocks, neurotypical participants underwent 15/25/15 trials, respectively, and TBI participants underwent 25/50/25 trials, respectively. More trials were undertaken for TBI participants since clinical collaborators had suggested more trials would be better elicit an effect in this population. For neurotypical participants, we followed trial-level procedures according to our previous work with this glove system (Liu et al., 2021). The three blocks of trials were repeated for each of two different feedback conditions during the training block: 1) *unimodal feedback* (audio cues only), 2) *multimodal feedback* (concurrent audio and visual cues). These two training conditions were presented in random order for each participant session completed within a single day.

### 2.4 Data analysis

All metrics were computed as trial averages for each participant before determining the effects of the participant group (TBI versus neurotypical) or feedback condition (unimodal or multimodal). Metrics for performance included the 3-D motion pathlength of the cubic object being transported and the task completion time (i.e., the time the object is being moved from initial to target positions). In both cases, performance is better when the metric is lower. Participants consistently placed the object at the target location; thus, accuracy measures were not evaluated. Instead, the primary performance metric was computed as the object’s motion pathlength while transported from initial to target locations, with completion time serving as a supplementary performance metric.

Neural activity was computed as EEG power in the alpha (8–12 Hz) and beta (13–30 Hz) bands. Additionally, EMG metrics were calculated as the overall mean amplitude across all seven muscles recorded and EMG-EEG coherence. EMG-EEG coherence was computed between an intrinsic hand muscle with the highest EMG amplitude for that participant group (abductor pollicis brevis for neurotypicals, abductor pollicis longus for TBI) and the EEG electrode corresponding to the M1 motor area. Different muscles expressing, on average, maximum EMG amplitude suggests some variation in grip strategy between the neurotypical and TBI groups. However, the variations in grip are likely negligible given abductor pollicis muscles are still highly recruited and likely will reflect changes in EMG-EEG coherence primarily based on feedback training conditions, as designed.

The mean EEG power overall (across all channels) and within the alpha and beta frequency bands were analyzed using “EEGLAB” in MATLAB^®^. Mean values for EEG and EMG were computed within a time window that spanned one second before the achievement of secure grasp to one second after the release of the object. All metrics were evaluated as a percentage change from “pre” to “post” blocks to assess the effects of training.

The Kolmogorov–Smirnov test was applied to confirm sufficient normality of each data set to be analyzed by a parametric test. A two-way ANOVA was applied on each measure to determine the effects of the two main experimental factors: feedback condition (i.e., unimodal: audio only; multimodal: audio + visual) and participant group (i.e., neurotypical, TBI). A paired two-sample *t*-test was used for assessing the simple effects of feedback conditions on each measure within participant groups since identifying the potential impact of feedback conditions for clinical populations is of primary interest in this study. In addition, a one-sample *t*-test was applied for each pairing of group and feedback condition to determine whether a significant post-training change occurred from baseline (i.e., a non-zero % change). Finally, linear regressions were applied to verify dependent relationships between performance and EEG and whether significant linear trends existed in trial-by-trial changes of each measure within (during) the training block.

## 3 Results

**FIGURE 3:**
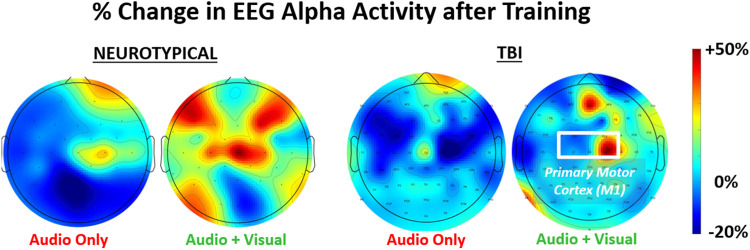
Representative relative change in regional brain activity (EEG alpha power) after feedback training of a grasp-and-place task. Results shown for neurotypicals and participants with traumatic brain injury (TBI) under two conditions of training with augmented sensory feedback (unimodal: audio only; multimodal: audio plus visual).

Examples of brain (alpha power) activation plots are shown for each group (neurotypical, TBI) paired with a training feedback condition (unimodal, multimodal) in Figure 3. Relatively higher alpha power is grossly observable with multimodal feedback for both groups; however, the activation regions appear more diffuse with neurotypicals. For multimodal feedback in TBI, two areas of concentrated activation are evident, including one near the primary motor cortex (M1).

**FIGURE 4:**
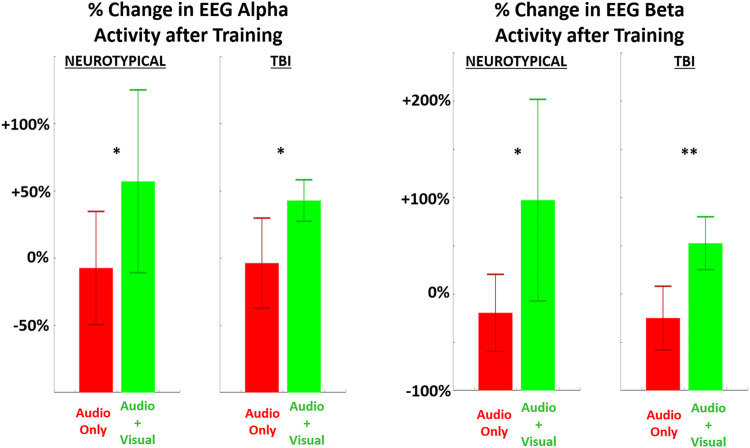
The relative (percentage) change in EEG power (LEFT = alpha band, RIGHT = beta band) in the performing grasp-and-place task is shown from before training (baseline) to after training with augmented sensory feedback. Results are compared between feedback conditions (unimodal: audio only; multimodal: audio plus visual) per participant group (neurotypicals, TBI). Note: *p < 0.05, **p < 0.01, ***p < 0.001 in comparing effects of feedback condition within each group.

The mean values of the percentage changes (i.e., from pre-to post-training) for each metric and the overall mean value at baseline (i.e., pre-training) are provided in Table 1. It should be noted that baseline values were not significantly different between neurotypical and TBI groups for any metric. In addition, for each measure, the specific *p*-values for individual comparisons between feedback conditions within each group (2-sample *t*-test), non-zero change from baseline (1-sample *t*-test), and aggregate factor-level effects for group and training feedback condition (2-way ANOVA) are shown in Table 2. From 2-way ANOVA, only the EEG and EMG-EEG coherence metrics showed a significant factor-level difference and only for the factor of training feedback condition. This result further highlighted the need to examine the simple effects of feedback on each metric within each group to affirm the critical hypothesis of this study (i.e., the presence of neurological dysfunction will alter how multimodal versus unimodal feedback impacts brain activity, muscle engagement, and functional performance of a motor rehabilitation task). Thus, individual metric results are discussed further within the context of feedback conditions with each participant group in the bar plots shown in Figure 4 through 6.

**TABLE 1:** Mean baseline (pre-training) value for each metric of interest and mean relative percentage change from baseline after (post) training.

Metric	Mean baseline NT (*units*)	Mean baseline TBI (*units*)	Mean % change from baseline							
			NT-A	NT-AV	TBI-A	TBI-AV	NT	TBI	A	AV
*EEG-alpha*	(3.3 ± 8.8) E03	(2.2 ± 7.2) E03	−7.4 ± 43	57 ± 68	−3.7 ± 33.5	43 ± 15	25 ± 64	20 ± 35	−6.1 ± 38	52 ± 55
*EEG-beta*	(1.1 ± 2.6) E04	(0.6 ± 2.0) E04	−20 ± 40	97 ± 104	−25 ± 33	53 ± 27	39 ± 97	14 ± 50	−22 ± 36	78 ± 82
*PERF-pathlength*	34 ± 4.2 (*cm*)	34 ± 4.3 (*cm*)	−19 ± 3.3	21 ± 7.8	22 ± 14	−21 ± 15	0.7 ± 21	0.7 ± 26	−4.7 ± 22	6.1 ± 23
*PERF-completion time*	1.5 ± 0.4 (*sec*)	1.3 ± 0.4 (*sec*)	−20 ± 14	−4.7 ± 15	18 ± 29	−12 ± 20	−12 ± 16	2.9 ± 29	−6.4 ± 27	−7.3 ± 17
*EMG-amplitude*	4.4 ± 2.3 (*mV*)	1.4 ± 4.2 (*mV*)	−7.3 ± 14	−1.6 ± 9.5	−21 ± 33	6.4 ± 37	−4.4 ± 12	−7.4 ± 36	−11 ± 20	0.6 ± 20
*EMG-M1 coherence*	0.25 ± 0.015 (*unitless*)	0.25 ± 0.020 (*unitless*)	3.8 ± 6.1	−4.3 ± 5.3	1.2 ± 4.5	−2.5 ± 5.5	−0.3 ± 7.0	−0.7 ± 5.2	2.9 ± 5.6	−3.7 ± 5.3

Note: Percentage changes reported per participant group (NT, neurotypical; TBI, traumatic brain injury) and per feedback condition [A, audio only (unimodal); AV, audio plus visual (multimodal)].

**TABLE 2:** *p*-values indicating if a post-training change in metric is significant (*p* < 0.05) in comparison to zero (1-sample *t*-test), between group-condition pairs (2-sample *t*-test), and within levels for each factor (2-way ANOVA, factors: participant group, feedback condition).

Metric	1-Sample				2-Sample		2-way ANOVA	
	NT-A	NT-AV	TBI-A	TBI-AV	NT-A vs. NT-AV	TBI-A vs. TBI-AV	Participant group: TBI vs. NT	Feedback condition: A vs. AV
*EEG-alpha*	0.57	**0.019 (*t* = 2.8)**	0.80	**0.001 (*t* = 6.8)**	**0.027 (*t* = -2.6)**	**0.025 (*t* = -3.2)**	0.76	**1.3E-03 (F = 12)**
*EEG-beta*	0.21	**0.034 (*t* = 2.6)**	0.12	**5.4E-03 (*t* = 4.7)**	**0.029 (*t* = -2.7)**	**0.0028 (*t* = -5.5)**	0.31	**3.0E-04 (F = 18)**
*PERF-pathlength*	**8.8E-11 (*t* = -21)**	**6.7E-07 (*t* = 9.4)**	**6.9E-03 (*t* = 4.0)**	**0.011 (*t* = -3.6)**	**4.9E-11 (*t* = --21)**	**1.8E-04 (*t* = 8.2)**	0.99	0.13
*PERF-comp. time*	**2.6E-04 (*t* = -5.1)**	0.29	0.15	0.16	**0.020 (*t* = -2.6)**	**0.030 (*t* = 2.8)**	0.042	0.89
*EMG-amplitude*	0.077	0.56	0.22	0.72	0.28	0.10	0.70	0.095
*EMG-M1 coherence*	**0.046 (*t* = 2.2)**	**0.017 (*t* = -3.0)**	0.52	0.27	**8.8E-03 (*t* = 3.1)**	0.10	0.83	**6.0E-04 (F = 14)**

Note: *p*-values < 0.05 are bolded with *t*-stat or F-stat included.

Significant post-training changes were observed in neurological activity (EEG power) within the alpha and beta bands after training with multimodal versus unimodal feedback (Figure 4). Again, results are expressed as the percentage change in each measure after training compared to before, i.e., at baseline. Neurological activity was significantly (*p* < 0.05) increased in both groups with multimodal feedback for both alpha and beta bands. This increase in activity with multimodal feedback is demonstrated as significant compared to unimodal feedback and from baseline. Unimodal feedback did not produce significant changes from the zero baseline (Table 2).

**FIGURE 5:**
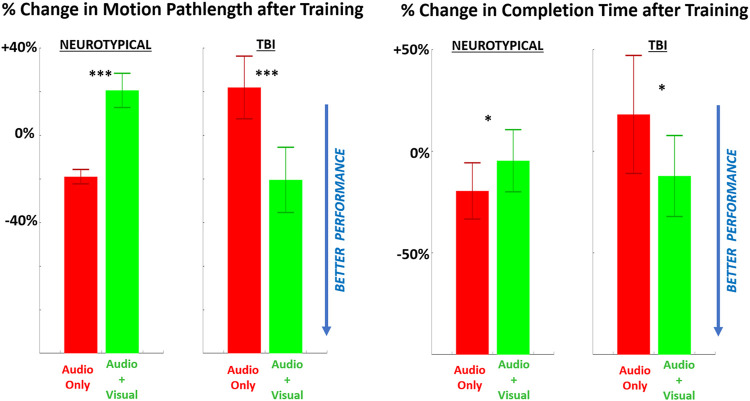
The relative (percentage) change in performance (LEFT = motion pathlength, RIGHT = completion time) in performing the grasp-and-place task is shown from before training (baseline) to after training with augmented sensory feedback. Results are compared between feedback conditions (unimodal: audio only; multimodal: audio plus visual) per participant group (neurotypicals, TBI). Note: *p < 0.05, **p < 0.01, ***p < 0.001 in comparing effects of feedback condition within each group.

For performance, both metrics showed improvement (i.e., shorter pathlengths, shorter completion time) with multimodal feedback, compared to unimodal feedback (Figure 5), for the TBI group. However, these performance trends were reversed (i.e., performance worsened with multimodal feedback) for neurotypicals. Of further note, the change in motion pathlength was significantly different from zero (baseline) for every pairing of group and training condition. The change in completion time was significantly non-zero only for neurotypicals with unimodal feedback with significantly improved (reduced) completion time compared to baseline.

**FIGURE 6:**
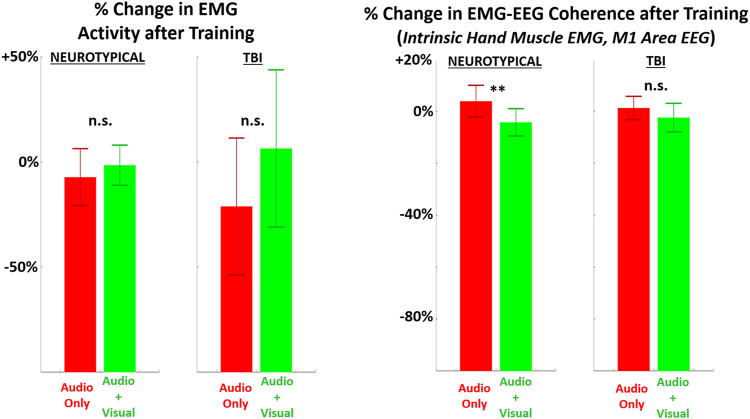
The relative (percentage) change in EMG metrics (LEFT = average EMG amplitude across all muscles recorded, RIGHT = EMG-EEG coherence between intrinsic hand muscle with highest amplitude and M1 brain area) in performing the grasp-and-place task is shown from before training (baseline) to after training with augmented sensory feedback. Results are compared between feedback conditions (unimodal: audio only; multimodal: audio plus visual) per participant group (neurotypicals, TBI). Note: *p < 0.05, **p < 0.01, ***p < 0.001 in comparing effects of feedback condition within each group.

EMG metrics did not demonstrate significant differences in any case for persons with TBI (Figure 6). However, there was a significant difference in EMG-EEG coherence between training feedback conditions for neurotypicals. Furthermore, for neurotypicals, the unimodal feedback condition produced a significant increase in EMG coherence from baseline after training, but multimodal feedback produced a significant decrease in coherence.

**FIGURE 7:**
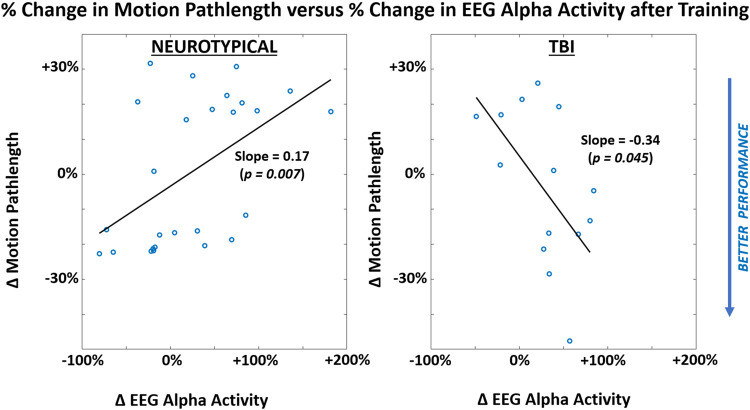
Correlations between participant-level mean values of performance metric (motion pathlength) and neural activity (EEG alpha power) within each participant group (LEFT = neurotypical, RIGHT = TBI). Results are shown for after training and pooled across both feedback conditions. Correlation represented through slope parameter for linear regression fitted to data. Slope magnitudes were assessed to be significantly non-zero (*p* < 0.05).

When attempting to discover a correlation between performance and neural activity, a significant non-zero slope parameter with linear regression was observed in relating motion pathlength to EEG alpha activity separately for each participant group across both conditions (Figure 7). Notably, the TBI group demonstrated improved performance (reduced motion pathlength) with increased EEG alpha power. However, the neurotypical group showed worsened performance with increased EEG activity.

**FIGURE 8:**
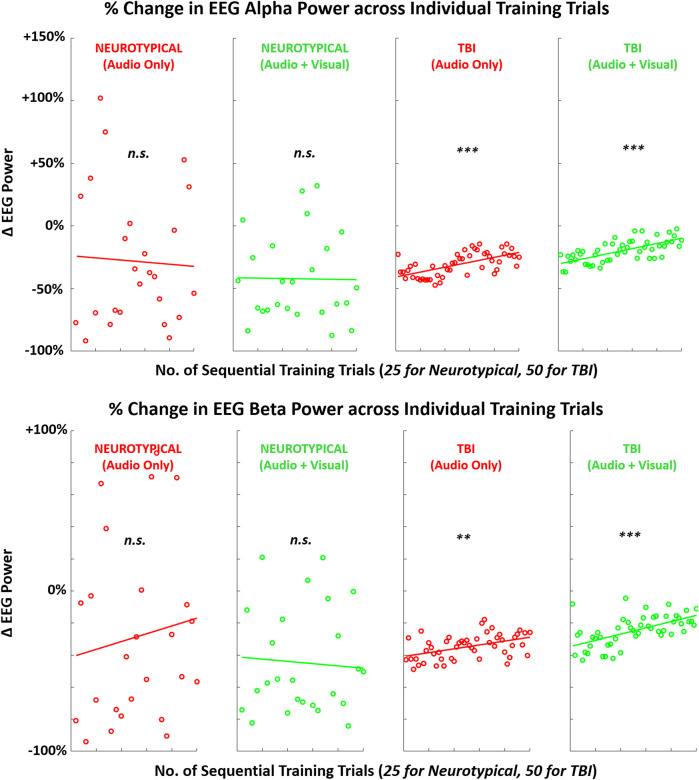
Mean (across participants) of EEG activity (alpha, beta) across sequence of training trials during training with augmented sensory feedback (audio or audio + visual) for each participant group (neurotypical or TBI). Linear regression fitted to indicate global trend within training block in each case pairing feedback condition and participant group. Note: *p*-values indicate non-zero value for slope coefficient (i.e., significant trend present) of linear regression; *p < 0.05, **p < 0.01, ***p < 0.001.

**FIGURE 9:**
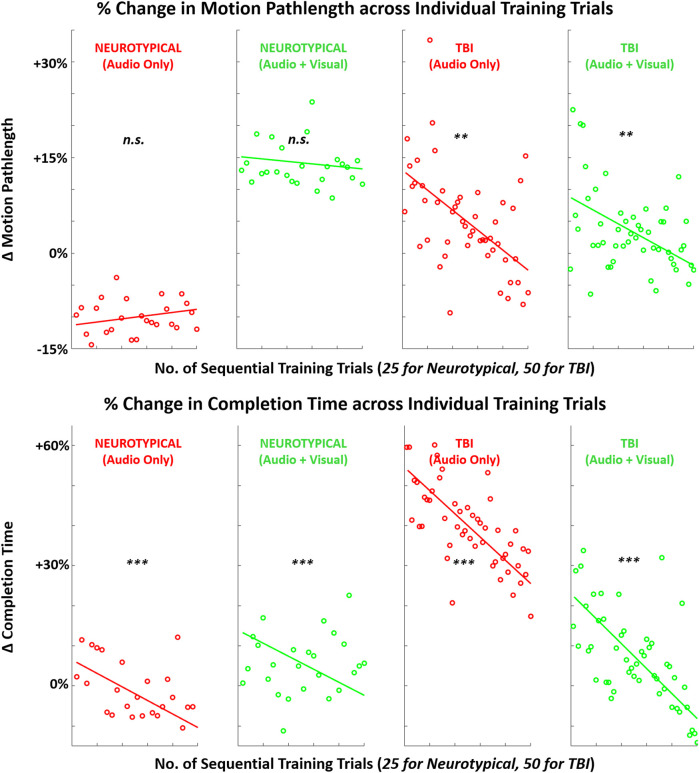
Mean (across participants) in performance metrics (motion pathlength, task completion time) across sequence of training trials with augmented sensory feedback (audio or audio + visual) for each participant group (neurotypical or TBI). Linear regression fitted to indicate global trend within training block in each case pairing feedback condition and participant group. Note: *p*-values indicate non-zero value for slope coefficient (i.e., significant trend present) of linear regression; *p < 0.05, **p < 0.01, ***p < 0.001.

**FIGURE 10:**
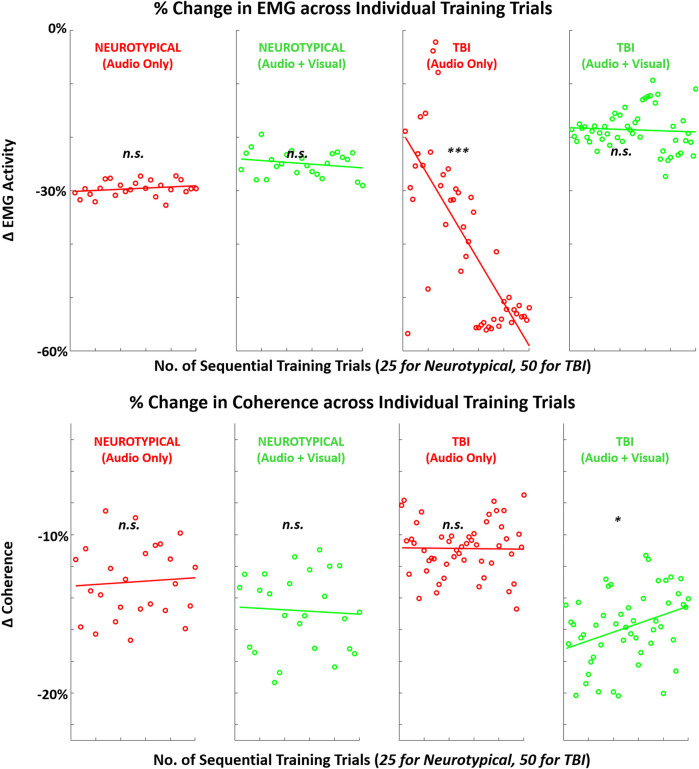
Mean (across participants) in EMG-related metrics (EMG amplitude, EMG-EEG coherence) across sequence of training trials with augmented sensory feedback (audio or audio + visual) for each participant group (neurotypical or TBI). Linear regression fitted to indicate global trend within training block in each case pairing feedback condition and participant group. Note: *p*-values indicate non-zero value for slope coefficient (i.e., significant trend present) of linear regression; *p < 0.05, **p < 0.01, ***p < 0.001.

When examining trends in each metric during training trials, at least one significant difference was observed for each metric pending the specific group or training condition. For both feedback conditions, EEG metrics significantly increased across training trials in the TBI group (Figure 8). For performance metrics (Figure 9), significant improvements (reductions) were observed in completion time for all four group-condition pairs. Significant improvements were observed in motion pathlength only for TBI but with both feedback conditions, leaving non-conclusive trends in motion pathlength for neurotypicals with both conditions. For EMG (Figure 10), a significant reduction in EMG amplitude was observed for TBI and audio-only feedback. In contrast, a significant increase in EMG-EEG coherence was observed for TBI, but with multimodal feedback. Training trends for all remaining EMG cases were inconclusive. The specific slope and associated *p*-values are presented in Table 3.

**TABLE 3:** The mean slope value (percentage change per trial repetition) during training block per group-condition pair and *p*-values indicating non-zero slope.

Metric	NT-A	NT-AV	TBI-A	TBI-AV
*EEG-alpha*	−0.34 (*p* = 0.86)	−0.06 (*p* = 0.95)	**0.39 (*p* = 3.3E-06)**	**0.41 (*p* = 1.0E-06)**
*EEG-beta*	0.97 (*p* = 0.57)	−0.28 (*p* = 0.75)	**0.24 (*p* = 2.6E-03)**	**0.39 (*p* = 7.9E = 05)**
*PERF-pathlength*	0.10 (*p* = 0.14)	−0.08 (*p* = 0.47)	**−0.31 (*p* = 1.2E-03)**	**−0.22 (*p* = 4.2E-03)**
*PERF-completion time*	**−0.67 (*p* = 5.0E-04)**	**−0.65 (*p* = 3.0E-04)**	**−0.58 (*p* = 1.4E-07)**	**−0.62 (*p* = 3.1E-03)**
*EMG-amplitude*	0.04 (*p* = 0.28)	−0.07 (*p* = 0.30)	**−0.80 (*p* = 1.1E-07)**	−0.02 (*p* = 0.72)
*EMG-M1 coherence*	0.02 (*p* = 0.75)	−0.02 (*p* = 0.80)	−1.8E-03 (*p* = 0.93)	**0.05 (*p* = 0.032)**

Note: *p*-values < 0.05 are bolded.

## 4 Discussion

This study evaluated how varying the nature of augmented sensory feedback used for motor training with virtual reality can impact post-training changes in neurological activity, motor performance, and muscular engagement for a grasp-and-place task. The central experimental factor was cueing neurotypical and TBI participants about secure grasp with either unimodal (audio cue only) or multimodal (audio cue plus visual cue) feedback during each training repetition. Ultimately, we examined the effects of training with each feedback condition by comparing EEG power, motor task performance, and EMG measures immediately after (post) training compared to before (pre) training, serving as the comparative baseline for each participant. Our primary finding was that the effects broadly observed on these measures were unique depending on whether participants were neurotypical or had moderate-to-severe TBI.

Both groups exhibited increased EEG activity, in both alpha and beta bands, after training with multimodal feedback compared to unimodal feedback. More robust EEG responses are generally expected following more exposure to sensory stimulation (Teplan et al., 2006). However, the relative increases with TBI were larger than neurotypicals and may have impacted respective motor outputs accordingly. With multimodal feedback, there were contradictory findings in performance as TBI participants significantly improved (reduced) their average motion pathlength and completion time; however, the additional visual cueing with multimodal feedback worsened performance in both metrics for neurotypicals. This seemingly paradoxical outcome across groups suggests a difference in how the added visual feedback is processed and leveraged for motor performance pending functional neurological states. In particular, multimodal feedback may support the expedited crossing of neural thresholds to improve learning as intended with multimodal feedback (Seitz and Dinse, 2007) for persons having TBI. Yet, for neurotypicals, the additional cueing may be excessive stimuli interpreted as confounding during task training and ultimately interferes with performance and learning progression (Spruit et al., 2016).

Such a finding suggests the need to optimize a computerized rehabilitation interface for users with neurological dysfunction. More specifically, guidance feedback may need to be delivered with greater sensory stimulation. The disturbed brain networks, such as after TBI, can alter how sensory feedback is processed for motor function (Nudo, 2013). Although not analyzed for significant differences, the brain plots in Figure 3 suggest the disparities in regional activation between TBI and neurotypical participants. Thus, increasing sensory stimulation with guidance feedback, even if redundantly encoding the same performance information, may partially compensate for processing dysfunctions with TBI. In any case, assessing the responses to feedback by neurotypicals independently from TBI participants is warranted. However, we still conducted a 2-way ANOVA to determine if each measure is broadly affected by each of the two main factors of group and feedback condition. Only the EEG measures (i.e., alpha power, beta power, and EMG-EEG coherence) demonstrated significant factor-level effects and only for feedback conditions.

While alpha activity is typically suppressed with active movements, it can reflect greater motor preparation (Deiber et al., 2012) and be enhanced by motor training paradigms that increase cognitive flexibility (Lasaponara et al., 2017). The post-training increase in alpha-band activity may suggest the foundation for early and robust consolidation of motor learning features from a pre-learning state (Henz and Schöllhorn, 2016). This phenomenon is readily shown with differential learning, characterized by practice variability to facilitate faster learning rates (Tassignon et al., 2021). The grasp-and-place task for this study was repetitive as it did not vary between trials; however, participants may perceive more variability when training with multimodal feedback. In the case of TBI, this perception of variability may be more effectively leveraged to improve potential motor learning.

On the other hand, beta activity can indicate increased alertness, including by visual stimuli (Kamiński et al., 2012), as done in this study. Regarding motor function, beta waves, especially over the motor cortex, are associated with strengthened sensory feedback during movement changes (Lalo et al., 2007). Thus, the long-term implications of increasing beta activity after each training session may facilitate learning through higher sensory-guided attention during movement training. While EMG metrics in this study were relatively insensitive to changes in feedback training, EMG-EEG coherence was significantly reduced for neurotypicals when receiving multimodal feedback. Since corticomuscular drive, especially in the beta band, indicates a change in muscle coordination strategy (Reyes et al., 2017), the attenuation of corticomuscular coherence may suggest that neurotypicals experienced divided attention (Johnson et al., 2011) in perceiving the added visual cue.

Although assessing post-training effects across measures of EEG, performance, and EMG from the pre-training baseline was the primary objective of this study, we also examined trial-by-trial trends during training for each measure. This analysis provides insight into how these measures may be actively manipulated with each training condition before participants return to independent (unguided) task performance. Although both neurotypicals and TBI demonstrated increased post-training neurological activity, only TBI demonstrated a linear trend towards increased neurological activity within the training block. There was a significant non-zero slope towards increased power across sequential training trials in both the alpha and beta bands.

These findings indicate that TBI participants may have a more immediate tendency to reformulate neural connections during training with augmented feedback. Neural reorganization to facilitate motor recovery is a crucial objective with motor rehabilitation training, and it is primarily expected with visually guided actions (Kantak et al., 2012). Comparatively, neurotypicals may be more limited in their capacity for neural plasticity for a relatively simple motor task. Furthermore, these training trends in neural activation were mirrored with the key performance metric of motion pathlength for both groups. Only TBI participants exhibited a significant trend in reduced pathlength (better performance) during training with more trial repetitions. Such correlates between brain activity and performance can be expected during motor sequence learning (Orban et al., 2010). While the difference in the number of training trials for each group may have impacted the magnitude of the post-training effect, the same trends, i.e., progressive changes in metrics (Figures 8–10), for TBI are readily apparent even halfway through the block of training trials.

For the secondary performance metric of completion time, both groups demonstrated significant trends in reduction across training trials with both feedback conditions. This finding suggests that augmented sensory feedback naturally incentivizes faster movements with more training repetitions. This finding is consistent with another study demonstrating that augmented feedback can impact movement times of reaching movements, irrespective of fixed task parameters (e.g., movement amplitude) (de Grosbois et al., 2015). In comparison, training trends with EMG activity were not as evident. Still, TBI participants did demonstrate a significant training trend in reduced EMG with unimodal feedback and increased EMG-EEG coherence with multimodal feedback. Thus, only the TBI participants appeared to progressively re-organize neural and motor activity during single-session training with augmented sensory feedback.

Furthermore, this study revealed a positive linear-level dependence between higher alpha activity and improved motor performance for TBI, despite a relatively small sample size. This finding suggests that designing rehabilitation paradigms to target increases in alpha activity during the training of persons with TBI may support better motor performance. Identifying and understanding such correlations open new pathways to optimize computerized rehabilitation. For example, control systems can be developed to adapt (personalize) more intelligently specific VR design elements, including feedback features and enhancement levels (e.g., the brightness of color and pitch of sound). The objective of such control systems would be to modulate neural rhythms in ways that are more likely to induce targeted plasticity and increased gains in function.

A presumed limitation of this study is the lack of a more fundamental control condition whereby participants would undergo no augmented feedback for an entire training block. However, the primary goal of this work was to examine the differential impact of multimodal feedback within VR. Thus, this study’s main limitation is that the scope of the evaluation is restricted to a single training session. Authentic learning, and gains in function, can only be ascertained with long-term assessments (e.g., tracking performance across multiple follow-up sessions). Furthermore, the margins of improvement with augmented training feedback we observed in the single session, although significant, likely would not produce a discernible change in performing activities of daily living. Still, short-term performance improvements (i.e., immediately after a single training session) can indicate this approach’s potential for motor learning (Reyes et al., 2017). Initially developed in (Liu et al., 2021), our training approach integrates the sense of agency with augmented sensory feedback cues. Since alpha power may be the primary neural oscillation in the sense of agency (Kang et al., 2015), our approach may leverage a cognitive-sensorimotor synergy in motor training. Furthermore, alpha activity at human M1 for task-specific involvement indicates the potential for rapid motor learning (Muellbacher et al., 2001). Thus, increased alpha activity and improved performance for the TBI group suggest the potency of multimodal VR feedback to promote neuroplasticity for more effective neuromotor rehabilitation.

## 5 Conclusion

This study demonstrates that training with agency-inspired augmented feedback in VR can significantly impact post-training neural activity, motor performance, and muscular engagement, depending on if the feedback is unimodal or multimodal. Furthermore, these effects can also depend on whether the person has a cognitive impairment (e.g., traumatic brain injury). A notably higher increase in alpha- and beta-band EEG activity after training, especially in brain regions associated with motor planning and execution, may offer an underlying neural explanation for improving motor performance. Thus, augmented feedback, particularly multimodal feedback, provided with VR is a promising approach for rehabilitating motor function after brain injury. Results from this study should motivate future investigations into optimizing the delivery of sensory-driven feedback from computerized rehabilitation interfaces aiming to maximize functional outcomes.

## Data Availability

The raw data supporting the conclusion of this article will be made available by the authors, without undue reservation.
